# A Role for Xanthurenic Acid in the Control of Brain Dopaminergic Activity

**DOI:** 10.3390/ijms22136974

**Published:** 2021-06-28

**Authors:** Omar Taleb, Mohammed Maammar, Christian Klein, Michel Maitre, Ayikoe Guy Mensah-Nyagan

**Affiliations:** Biopathologie de la Myéline, Neuroprotection et Stratégies Thérapeutiques, INSERM U1119, Fédération de Médecine Translationnelle de Strasbourg (FMTS), Université de Strasbourg, CRBS 1, Rue Eugène Boeckel, 67000 Strasbourg, France; omar@unistra.fr (O.T.); mohammed.maammar@merckgroup.com (M.M.); c.klein@unistra.fr (C.K.)

**Keywords:** xanthurenic acid, kynurenic acid, dopamine, cognitive dysfunction, schizophrenia

## Abstract

Xanthurenic acid (XA) is a metabolite of the kynurenine pathway (KP) synthetized in the brain from dietary or microbial tryptophan that crosses the blood-brain barrier through carrier-mediated transport. XA and kynurenic acid (KYNA) are two structurally related compounds of KP occurring at micromolar concentrations in the CNS and suspected to modulate some pathophysiological mechanisms of neuropsychiatric and/or neurodegenerative diseases. Particularly, various data including XA cerebral distribution (from 1 µM in olfactory bulbs and cerebellum to 0.1–0.4 µM in A_9_ and A_10_), its release, and interactions with G protein-dependent XA-receptor, glutamate transporter and metabotropic receptors, strongly support a signaling and/or neuromodulatory role for XA. However, while the parent molecule KYNA is considered as potentially involved in neuropsychiatric disorders because of its inhibitory action on dopamine release in the striatum, the effect of XA on brain dopaminergic activity remains unknown. Here, we demonstrate that acute local/microdialysis-infusions of XA dose-dependently stimulate dopamine release in the rat prefrontal cortex (four-fold increase in the presence of 20 µM XA). This stimulatory effect is blocked by XA-receptor antagonist NCS-486. Interestingly, our results show that the peripheral/intraperitoneal administration of XA, which has been proven to enhance intra-cerebral XA concentrations (about 200% increase after 50 mg/kg XA i.p), also induces a dose-dependent increase of dopamine release in the cortex and striatum. Furthermore, our in vivo electrophysiological studies reveal that the repeated/daily administrations of XA reduce by 43% the number of spontaneously firing dopaminergic neurons in the ventral tegmental area. In the substantia nigra, XA treatment does not change the number of firing neurons. Altogether, our results suggest that XA may contribute together with KYNA to generate a KYNA/XA ratio that may crucially determine the brain normal dopaminergic activity. Imbalance of this ratio may result in dopaminergic dysfunctions related to several brain disorders, including psychotic diseases and drug dependence.

## 1. Introduction

**Figure 1:**
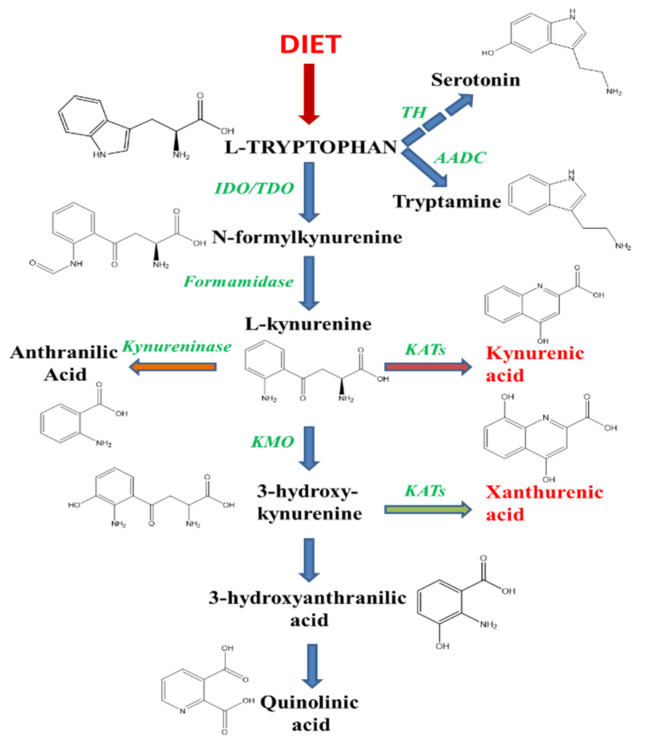
Tryptophan as a precursor of serotonin, XA and tryptamine. Tryptophan is an essential amino acid the concentration of which is regulated by food intake and gut microbiota. Tryptophan and many of its metabolites are transported into the brain via regulated mechanisms and give rise to many active compounds in the CNS. The principal pathway is degradation through kynurenine and the synthesis of two related analogues, kynurenic and xanthurenic acid. These two compounds have opposite effects on dopamine disposal in the brain. In addition, tryptophan is the precursor of the serotonin neurotransmission pathway and of tryptamine, an indolamine compound which stimulate trace-amine receptors in the CNS, especially TAAR1. The three pathways of tryptophan degradation produce substances linked to psychosis and are involved in the modulation of dopaminergic activities in the brain. TH, tryptophan hydroxylase; AADC, aromatic amino-acid decarboxylase; IDO, indolamine dioxygenase; TDO, tryptophan dioxygenase; KATs, kynurenine aminotransferases; KMO, kynurenine monooxygenase.

Under physiological conditions in the mammalian brain, about 95% of the essential amino-acid tryptophan available from the diet is metabolized through the kynurenine pathway (KP, Figure 1) in competition with the formation of serotonin and melatonin via 5-hydroxytryptophan (5-HTP) synthesis [1] and the decarboxylation of tryptophan into tryptamine. The KP leads to the formation of several neuroactive compounds, some of which (including 3-hydroxykynurenine (3-HK), 3-hydroxyanthanilic (3-HAN) and quinolinic acid (QUIN)) are neurotoxic while other metabolites, including kynurenic acid (KYNA), have neuroprotective properties [2,3,4]. A parent compound of KYNA, structurally related, is xanthurenic acid (XA). Both compounds result from the transamination by kynurenine aminotransferases (KATs) of either L-kynurenine for KYNA or 3-HK for XA [5,6,7]. The balance between KYNA and XA is mainly due to the activity of kynurenine monooxygenase (KMO), whose inhibition increases the concentration of KYNA and in theory decreases the production of XA [8,9,10].

Many studies have been devoted to the identification of the neurophysiological role of KYNA which negatively targets NMDA and α7 nicotinic receptors and is principally produced in astrocytes because of KAT II activity [11,12,13]. Several pathological conditions have been associated with a disturbance of the KP and specifically with anomalies of KYNA synthesis [6,7,14]. Among these pathological problems, studies from schizophrenic patients suggest that negative symptoms and cognitive deficits can be associated with a reduction of dopamine release in frontal cortex [15,16]. A systematic review has confirmed that KYNA levels increase in the cerebrospinal and brain tissue of schizophrenic patients [17,18,19]. Therefore, based on the dopaminergic hypothesis of this disease, particularly the role of D2 midbrain receptors, the inhibitory effects of sub-micromolar amounts of KYNA on striatal dopamine release have been suggested as potentially involved in the pathophysiological mechanisms of the disease [15,17,20,21,22]. However, while the balance between KYNA and XA appears pivotal in the modulation of neurobiological functions, the role of XA in the regulation of brain dopaminergic activity remains unclear. Indeed, both KMO and KAT II activities give raise to XA, a close structural analogue of KYNA which is present in neurons [23]. KAT II appears to have the major role in the synthesis of XA from 3-HK in the brain [5]. The neuronal localization of this enzyme has been described recently in the cerebellum [24]. XA distribution in the brain, its transport and release suggest a role for this compound in synaptic transmission [25]. XA binds to a neuronal GPCR [26], decreases extracellular glutamate levels by interacting with vesicular transport [27] and activates group II metabotropic glutamatergic receptors [28,29]. Its putative role in the pathophysiology of schizophrenia is supported by its reduced brain and serum levels in patients [30,31]. Altogether, these data prompted us to investigate the effect of XA on brain dopaminergic activity. Therefore, here we combined various approaches, including microdialysis infusion of XA, dopamine quantification using high performance liquid chromatography coupled with electrochemical detection, stereotaxic method and in vivo electrophysiological recording of dopaminergic neurons to determine the pharmacological action of XA on dopamine release and dopaminergic activity in the rat brain. 

## 2. Results

To assess the potential role of XA in the control of DA release at the prefrontal level (meso-cortical pathway), we used two in vivo approaches to evaluate the acute and the chronical effects of XA on DA neuron activity. Acute XA effects were assessed by micro-dialysis, measuring the prefrontal cortex extracellular content of DA following local applications of XA. Chronic XA effects were tested using electrophysiological recording of DA neuronal spontaneous firing in the VTA and SN nuclei after repeated treatment of the animals with XA.

### 2.1. XA Retro-Dialysis Induces an Increase in Dopamine Extracellular Concentration in the Rat Prefrontal Cortex

**Figure 2:**
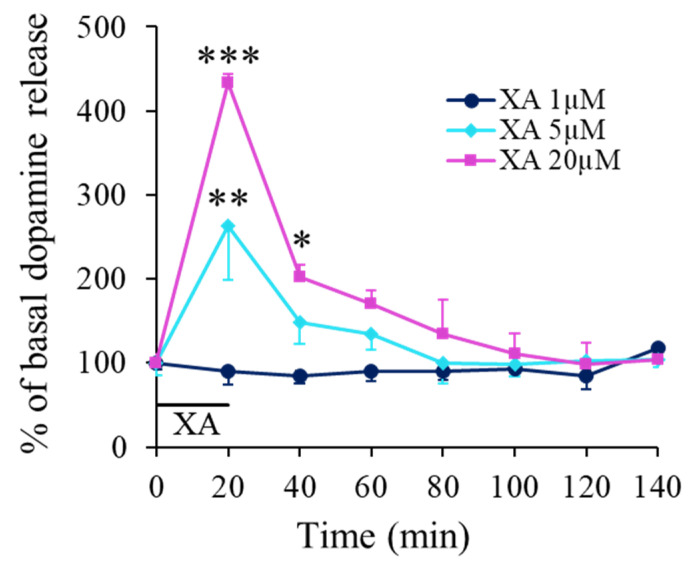
XA-induced DA release in the prefrontal cortex. A. Time evolution of DA release measured by microdialysis. Filled symbols represent the mean percentage (±SEM) of the mean basal DA release (mean of 8 × 20 min dialysate fractions before retro-dialysis of XA) measured in consecutive 20 min cumulated dialysate. Continuous lines are interpolation between data points. The black bar indicates the period of XA application at concentration of 1, 5 or 20 µM as indicated. The dopamine basal release level (100%) was 433 ± 32 fmol/20 min for the 20 µM-treated group (*n* = 3 to 5 rats per group). Under the influence of XA administered directly to the brain tissue, we observed a graduated increase in the extracellular dopamine with a local four-fold increase by reference to controls for a concentration of XA of 20 µM. The local diffusion of XA into the brain through the probe was estimated in vitro around 18%. * *p* < 0.05; ** *p* < 0.01; *** *p* < 0.001, comparison with control before XA infusion. Not corrected from in vitro recovery.

**Figure 3:**
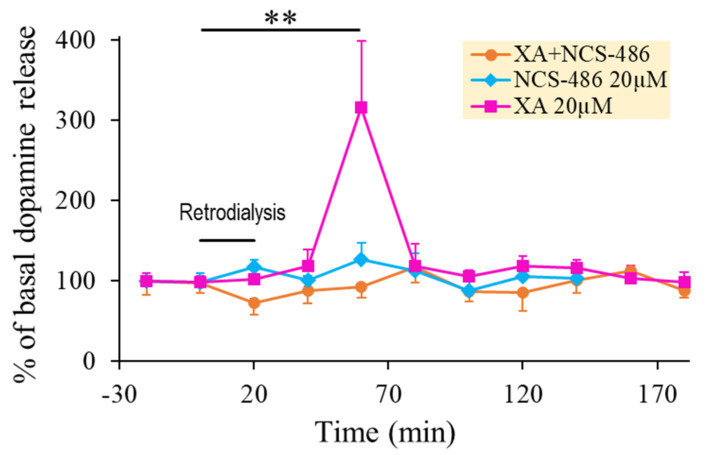
XA-induced DA release was blocked by NCS-486. A. Time course of the effect of 20 µM XA local application on DA release obtained in the absence or presence of 20 µM of the XA-receptor antagonist, NCS-486, as indicated. Data points are mean percentage ±SEM of the mean basal release of dopamine (mean of 8 × 20 min dialysate fractions before drug perfusion) and lines are interpolation between data points. The black horizontal bar indicates the period of local 20 min application by retro dialysis of 20 µM XA, co-infusion of XA 20 µM + NCS-486 20 µM or 20 µM of NCS-486 alone as indicated (*n* = 3 animals per group). Mean dopamine basal release (100%) was 130 ± 23 fmol/20 min. The infusion of XA alone induces a strong dopamine response, while the presence of NCS-486 completely blocked this dopamine increase. NCS-486 alone has no significant effect. ** *p* < 0.01 comparison with control before retro dialysis.

Local application of XA through the microdialysis probe affected dose-dependently the dopamine (DA) concentration in the prefrontal cortex extracellular space. Indeed, at the lowest XA concentration (1 µM) tested, no significant change in the DA release was observed (Figure 2). In contrast, at concentrations of 5 and 20 µM, XA induced a rapid DA concentration increase that peaked at about 20 min giving average peak values of 264 ± 64 and 434 ± 10% of basal DA release, respectively. The recovery kinetics to basal DA levels (100%) were relatively slow and 20 min after the end of drug application, the mean increase in extracellular dopamine obtained with 20 µM XA is still twice the amount observed for 1 µM and full recovery was seen about 80 min after the peak (Figure 2). This kinetic had however some variability, and in some experiments, as in Figure 3, the response was more transient with a delayed peak and a faster recovery.

### 2.2. The Effect of XA Local Application on Dopamine Release in the Prefrontal Cortex Was Blocked by the Antagonist NCS-486

NCS-486 is a structural analogue of XA synthesized in the CNRS UMR-7200 lab, Faculty of Pharmacy, Strasbourg University, France. This compound has been described in a previous publication [26]. It competes with XA binding on rat brain synaptosomal membranes with an IC_50_ of about 14 µM and possesses antagonistic properties at the XA receptor, as demonstrated by electrophysiological studies on NCB-20 neuroblastoma cell line [26]. Figure 3 shows the interaction of XA and NCS-486 on DA release. While NCS-486 20µM by itself did not affect basal DA release, when coapplied with XA 20 µM, it fully blocked XA-induced DA release. 

### 2.3. Doses-Effect of XA Administration on Frontal Cortex and Striatum Dopamine Contents

**Figure 4:**
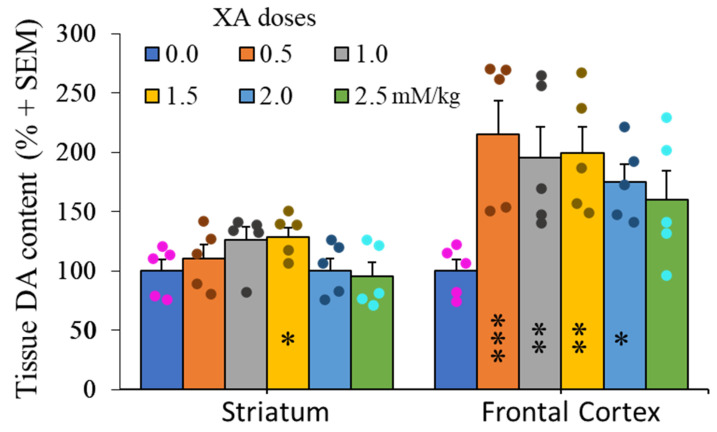
Dose-effect of XA administration on dopamine concentrations in the frontal cortex and striatum. Histogram of mean (+SEM) DA tissue content as a function XA doses as indicated. Rats were sacrificed 60 min after i.p injection of XA. As previously described [25], the i.p. administration of 50 mg/kg of XA to the rats induced a strong increase in the brain concentration of this compound (frontal cortex and caudate putamen in particular). The figure shows a two-fold increase of DA concentrations in the frontal cortex after 0.5 mM/kg and these concentrations remain stable at higher doses of XA. By contrast, the change of DA-tissue content in the striatum is very low and almost not significant, even at the highest concentration of XA. Filled circles represent individual data point sets for each bar of the histogram accordingly. * *p* < 0.05; ** *p* < 0.01; *** *p* < 0.001; post ANOVA comparison with control animals using the uncorrected Fisher’s LSD statistical test.

XA administration to rats via intraperitoneal route has been demonstrated to significantly increase the in vivo concentration of this compound in several regions of the brain, including frontal cortex and striatum [25]. Figure 4 shows the effect of increasing doses of i.p injected XA on dopamine tissue content in the rat brain. In the frontal cortex, 0.5 mM/kg of XA increased dopamine content by about two times. This level cannot be exceeded by increasing the amount of administered XA. Under the same conditions, XA did not induce more than about 30% increase of dopamine tissue content in the striatum.

### 2.4. Kinetics of Dopamine Variation in the Frontal Cortex and Striatum after Peripheral Administration of XA

**Figure 5:**
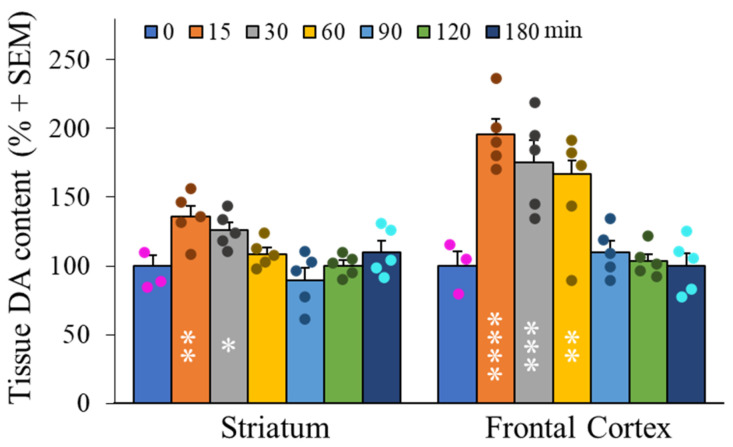
Time course of dopamine contents in frontal cortex and striatum after administration of XA (1 mMol/kg, i.p). A. Histogram of time evolution of mean (+SEM) DA tissue content, as indicated. The increase in DA was maximal at 15–30 min after administration of XA, then decreased gradually to control levels after 90 min. The increase of DA is particularly high in the frontal cortex. Filled circles represent individual data point sets for each bar of the histogram accordingly. * *p* < 0.05; ** *p* < 0.01; *** *p* < 0.001; **** *p* < 0.0001; post ANOVA comparison with control animals using the uncorrected Fisher’s LSD statistical test.

Following acute administrations of XA via intra-peritoneal route, modifications of dopamine contents were measured by extraction of rat frontal cortex and striatum as described previously [25]. Figure 5 shows the effect of XA injection as a rapid dopamine concentration increase in these two brain regions that peaked at about 20 min post-injection. This DA increase attained at the peak level mean values of about 100% in the frontal cortex and 30% in the striatum. Basal levels were recovered after 90 min. 

### 2.5. XA Repeated Treatment Reduced Spontaneous Firing in VTA but Not in SN

**Figure 6:**
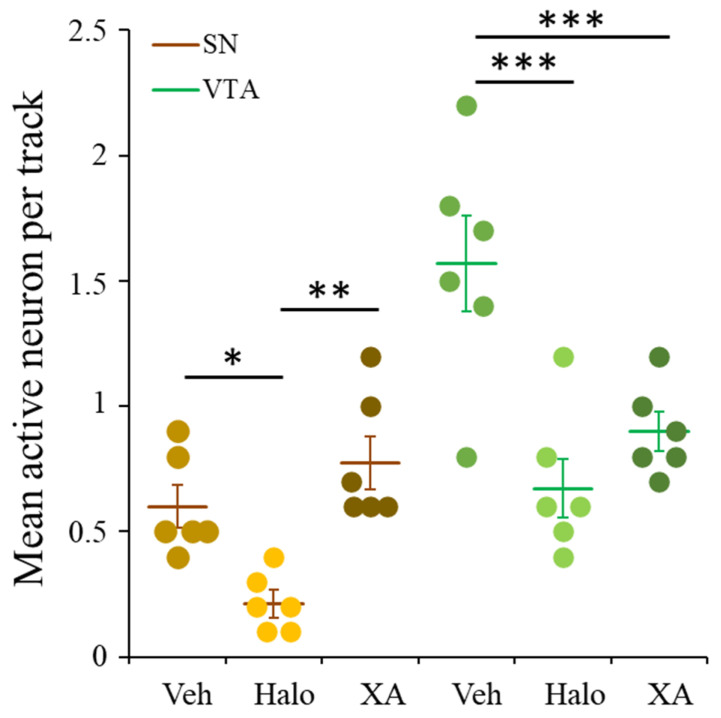
XA and haloperidol chronic effect on VTA and SN spontaneous neuronal firing. Three groups of six rats were daily i.p injected with vehicle, haloperidol (0.5 mg/kg) or XA (100 mg/kg) in 1 mL volume, respectively for 21 days and recorded at D22 in the conditions given in the methods section. Each of the data point (circles) represents the average number of active neurons found in the tracks explored per rat for left and right SN or VTA, respectively. Bar chart histogram of the mean (±SEM) data for the six rats per condition. * *p* < 0.05; ** *p* < 0.01; *** *p* < 0.001 Statistical significance obtained with one-way ANOVA followed by Newman-Keuls multiple comparison test for VTA. For SN data, since the Veh-group did not pass the normality test, the non-parametric statistical Mann-Whitney test was used to compare the Halo-group to the Veh- and XA-group.

To explore the effect of chronic XA administration on the dopamine activity in the meso-cortico-limbic and nigrostriatal pathways, the spontaneous firing of dopamine neurons was explored in the VTA and SN. Since the role of these two dopaminergic pathways have been implicated in psychotic symptoms, the effect of chronic XA was compared to the effect of chronic administration of the typical antipsychotic drug, haloperidol. Results of these comparative treatments are shown in Figure 6, by reference to rats treated with vehicle alone.

Spontaneous action potentials (AP) from VTA or SN neurons were biphasic or triphasic with duration of 2–3 ms (Figure S2). In control conditions (animal treated with vehicle) the mean active neurons per track was 1.6 ± 0.2 and 0.6 ± 0.1 for VTA and SN, respectively (for detailed data see Figure S3). XA 100 mg/kg/day or haloperidol 0.5 mg/kg/day treatment reduced significantly (one-way ANOVA followed by Newman-Keuls multiple comparison test, *p* < 0.001 as compared to control) the number of active neurons per track in the VTA and mean values of 0.9 ± 0.1 and 0.7 ± 0.1 active neuron were obtained, respectively. In contrast, XA treatment did not change the number of firing neurons in the SN, whereas haloperidol treatment decreased it significantly (*p* < 0.05 as compared to control). The mean value per track obtained with XA was 0.8 ± 0.1 firing neuron, that is similar to the control and significantly (*p* < 0.01) higher than that obtained with haloperidol (0.2 ± 0.1 active neuron per track). 

## 3. Discussion

XA and its structurally related compound KYNA are metabolites of the kynurenine pathway [32]. Their respective synthesis depends principally on the bioavailability of tryptophan, the inducible activity of IDO and TDO by inflammatory cytokines, and on the activity of KMO (kynurenine monooxygenase) for the synthesis of XA [33]. Both compounds are controlled by KATs II, whose expression was shown in glial cells [5] and in neurons [24]. However, specific antibodies directed against XA argue for an exclusive localization of this compound in neurons, in somato-dendritic endings [23]. Tyrosine hydroxylase, the rate-limiting enzyme of dopamine synthesis, is juxtaposed to XA-immunoreactive material but not co-localized with it. Previous studies have shown the heterogeneous distribution of XA in the rat brain together with its vesicular accumulation, transport in neuronal cells and Ca^2+^-dependent release that support a signaling role for this compound [25]. XA binds to crude synaptosomal membranes of rat brain with a Kd of 0.74 µM [26] which is close to the concentration of XA administered by reverse dialysis in the present study, considering the in vitro recovery measured for this substance through the dialysis membrane (18 ± 4%). After intra-peritoneal administration, XA enters the brain and significantly increases its endogenous concentration in most regions of the rat brain, including frontal cortex, mesencephalic nuclei A9–A10 and striatum [25,34]. This compound has also been suggested to inhibit vesicular glutamate transporters and to directly activate mGlu2 and mGlu3 receptors in heterologous models [27,28,29,35,36].

Our present data show that the local administration in brain of low micromolar amounts of XA increases the extracellular concentration of dopamine in frontal cortex and striatum, two brain regions linked to the meso-cortico-limbic and nigrostriatal dopaminergic pathways. This release is blocked by the XA receptor(s) antagonist NCS-486 at a dose compatible with its IC_50_ for the receptor. In addition, peripheral administration of XA significantly increases the tissue concentrations of XA (Gobaille et al., 2008) and dopamine in the two brain regions investigated. The chronic increase in XA concentration in the VTA and SN nuclei decreases the dopaminergic firing of neurons, for VTA similarly to what is also observed for the typical neuroleptic haloperidol, while for SN the spontaneous discharge of dopamine neurons was preserved with XA by contrast to haloperidol. Our results show that the chronic effect of XA on VTA corresponds to a 43% (compared to a 56% Halo-induced reduction) decreased proportion of spontaneously firing neurons in the dopaminergic neuron population and could lead to an important drop of the basal dopamine release at their projection sites, including the mesolimbic pathway.

Interestingly, comparison of the effect of XA on dopaminergic activity with the reported activity of acute KYNA on extracellular dopamine levels appears to be opposite [21,22,37]. KYNA, coming from astrocytes, is an antagonist of NMDA and α7 nicotinic receptors. It is synthesized from kynurenine while XA comes from the transamination of 3-hydroxykynurenine [5,12]. The activity of kynurenine monooxygenase (KMO) regulates the ratio between KYNA and XA. The inhibition of KMO increases the brain concentration of KYNA and probably decreases the level of XA [15]. Indeed, KMO inhibition or deletion should theoretically cause KYNA elevation and the decrease of 3-HK and subsequent metabolites [38]. KYNA, contrary to XA, dose-dependently reduced extracellular dopamine levels by more than 50% at a concentration of 0.5 µM. This result was obtained by in vivo microdialysis of the rat striatum, KYNA being administered by perfusion through the probe [22]. KMO inhibition by Ro 61-8048 induced an increase in KYNA concentration and a similar reduction of extracellular dopamine levels [37]. KMO appears to be a pivotal enzyme in the kynurenine pathway to regulate the synthesis of KYNA versus the production of XA in brain. Modification of its activity modulates the ratio KYNA/XA because it appears that kynurenine aminotransferase (KATs, especially KATs II) are not specific for the synthesis of either of these compounds. However, KYNA is released by astrocytes while XA has been reported to be localized in neurons [23,39]. These different cellular localizations might contribute to their different functional role on extracellular dopamine levels.

In our hands, chronic administration of the typical neuroleptic haloperidol (0.5 mg/kg during 21 consecutive days) induced a significant reduction of the dopaminergic neuron firing in the VTA and the SN compared to rats treated with vehicle. Interestingly, while chronic KYNA increase in KMO K/O mice, exhibiting a marked increase in spontaneous activity of VTA dopamine neurons [15], the chronic administration of XA induced the opposite effect. Indeed, the firing of dopamine neurons in the VTA was significantly reduced as compared to control animals, but the SN dopamine activity remains not affected. Therefore, it appears that KYNA effects on extracellular dopamine are opposite to that of XA both in acute or chronic conditions, and the regulated balance between the synthesis of these two compounds participate in the modulation of dopaminergic activity in the brain. XA and KYNA have both been suggested to be implicated in the pathophysiological mechanism of schizophrenia [40] and considering the role of the dopamine hypothesis in the etiology of the disease [17,41], the present work supports a role for the kynurenine pathway activity in psychotic pathology and cognitive dysfunctions. Our present findings support a much important role for XA in the regulation of dopaminergic activity in the frontal cortex compared to the striatum. The traditional role of the mesolimbic system in the pathophysiology of schizophrenia is now being discussed and dopaminergic dysfunctions are greatest within the nigrostriatal pathways implicating the dorsal striatum [20]. However, the present results only provide indications that XA participates in the regulation of dopamine accumulation and release in a different way according to the two brain structures explored.

In conclusion, our results are additional evidence to support an important role of tryptophan metabolites in the regulation of psychotic disturbances and/or addiction under the control of GPCRs stimulated by XA or TAAR1 (isoform 1 of the family of receptors termed trace-amine associated receptors [42]) modulated by tryptamine. XA and tryptamine which depend on tryptophan bioavailability and channeling through their respective pathways participate in dopaminergic neurotransmission and may therefore contribute with serotonin to the modulation of several physiological, behavioral and neuro-pathological functions which are involved in psychosis symptoms. With regards to dopamine regulation and functions, the trace amine tryptamine and XA, both deriving from microbiota, food and the same precursor tryptophan, are present in the brain at sub-micromolar amounts and interact with specific GPCR’s. The balanced and regulated production of these two trace compounds synthetized or transported to the brain remains unknown but may have a great potential interest in many behavioral and pathological events driven by the dopaminergic signaling (see for example Figure S4A,B). 

## 4. Materials and Methods

### 4.1. Animals

Adult male albino Wistar rats weighing 250 to 275 g, bred in the Faculty de Medicine (Strasbourg, France), were used for the experiments. The rats were housed paired in individual plastic cages (40 × 25 × 25 cm) on a standard 7 a.m. to 7 p.m. light/dark cycle, with free access to food and water. Animal procedures were conducted in accordance with guidelines of the European Community Council directive of 24 November 1986 (86/609/EEC). In addition, all experiments were performed minimizing the number of animals used and their suffering in accordance with the Alsace Department of Veterinary Public Health Guide for the Care and Use of Laboratory Animals (Agreement number E-67-482-35). A national project authorization was delivered by the French Ministry of Higher Education and Research and by CREMEAS (a local ethical committee, project authorization number APAFIS#9373-201605111128746v2). 

### 4.2. Materials

**Figure 7:**
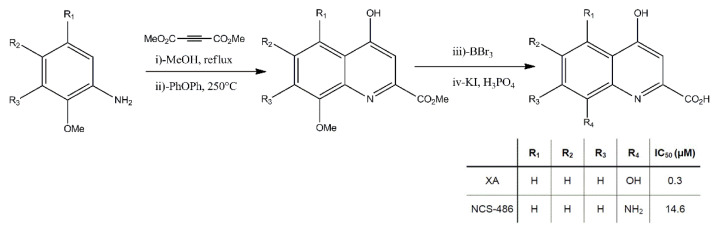
The synthesis of NCS-486 was carried out in a two-step reaction starting from readily available methyl-8-nitro-4-oxo 1,4 dihydroquinoline 2-carboxylate as described in [30]. Reduction of the nitro group and hydrolysis of the methyl ester were performed according to the previous description in Taleb et al. [26].

Xanthurenic acid was obtained from Sigma and NCS-486 was synthesized by the Faculty of Pharmacy, Strasbourg, France (Figure 7). 

All drugs were dissolved in the Ringer solution (final pH 7.0) or in sterile NaCl 0.9%.

### 4.3. Surgical Procedures

An I-shaped canula (CMA12 Carnegie, Sweden) was used for the experiments, with and exposed tip length of 4 mm. The dialysis membrane (OD 500 µm) was polycarbonate-polyether co-polymerized with a 20 KDa cut-off. For the implantation of the guide canula, rats were placed in a stereotaxic frame (Narishige) under ketamine chlorhydrate (Imalgène 500 Merial) anaesthesia (150 mg/kg i.p). Stereotaxic coordinates from the Bregma for the PFC were AP: +3; ML: +1.8; DV: −5.5 mm with a right angle of 15° in the coronal plane according to the Atlas of Paxinos and Watson [43]. 

### 4.4. Microdialysis Procedures

The experiments were performed in lesioned or non-lesioned conscious rats 24 to 48 h after surgery. All microdialysis experiments were undertaken between 9.00 a.m. and 6.00 p.m. The composition of the perfusion medium was the following: NaCl 147 mM; CaCl_2_ 1.2 mM; MgCl_2_ 1.2 mM and KCl 4.0 mM, pH 6.5. The flow rate was set at 1 µL/min with a microinjection pump (CMA 100, Carnegie). Dialysates were collected every 20 min and immediately stored in liquid nitrogen (−180 °C) till analysis. 

### 4.5. Dopamine Analysis

Dopamine was quantitated by high performance liquid chromatography (HPLC-EC) with electrochemical detection (Figure S1).

The chromatographic system consisted of a 25 cm × 4.6 mm Hypersyl C18 ODS column (particle size 5 µm, Biochrom, France). The column was kept at a constant temperature of 30 °C with a heating block (Waters model TCM), and the flow rate was 1.2 mL/min (Waters model 501 HPLC pump) with a back pressure of 1500 psi. The system was linked to a Waters model 460 electrochemical detector with a glassy-carbon electrode. The detector potential was maintained at 0.85 V versus an Ag/AgCl reference electrode. The mobile phase consisted of 0.05 M NaH_2_PO_4_ and 0.1mM disodium EDTA (pH 4.85) in double-distilled water with methanol added to a final concentration of 6%. The system was calibrated by injection of various amounts (0.2 nmol–20 fmol) of standard solutions containing dopamine and 5 pmol of internal standard. The whole dialysate (20 µL) of each sample was injected onto the column, and peak identification was performed by comparison of retention times regarding the calibration solutions.

### 4.6. In Vitro Recovery Experiments

To estimate the recovery of dopamine through the membrane, dialysis probes were immersed in 300 µL × 10^–6^ M dopamine, dissolved in the Ringer solution. The in vitro recovery of the membranes was for DA: 16% ± 2 (*n* = 6), at room temperature and a flow rate of 1 µL/min. For XA retro-dialysis experiments, the in vitro recovery was measured at 18% ± 4 (*n* = 6).

### 4.7. Histology

At the end of the experiments, the rats were sacrificed by an anaesthetic overdose of Ketamine chlorhydrate. Their brains were removed and stored in buffered formalin for at least 2 days. The correct placement of the microdialysis probe was verified by microscopic examination of the sections. 

After dialysis, the brains were removed and the correct placements of the microdialysis probe and the electrode were verified. The left and right striatum were dissected and stored in liquid nitrogen till analysis for dopamine tissue content. 

### 4.8. Electrophysiological Recording

#### 4.8.1. Animal Treatments

Animals (6 rats per condition) were treated during 21 consecutive days with Xanthurenic acid (XA; 100 mg/kg/day; dissolved in 1% bicarbonate solution). The drug was i.p injected in a 1 mL volume. The XA effect was compared to that of the neuroleptic haloperidol (0.5 mg/kg i.p in 1 mL) given daily for 21 days. Control animals were daily i.p injected with 1 mL of vehicle for the same period. 

After the treatment schedule (i.e., on day 22) spontaneously firing dopaminergic neurons within the SN and the VTA nuclei were counted. 

#### 4.8.2. Surgical, Stereotaxic, and Recording Procedures

The animals were anesthetized with ketamine (100 mg/kg i.p) and mounted on a stereotaxic apparatus (TSE system, Germany). During the experiment supplemental doses of anesthetic were administered if necessary. The skull was revealed by incision of the scalp and remaining flesh removed using cotton sticks. Left and right skull areas overlying the SN and VTA were removed. The following stereotaxic coordinates were used, relative to bregma and cortical surface: SN: AP = −5.4 to −5.8, ML = 1.8 to 2.5, DV = −7.0 to −8.0 and VTA: AP = −5.4 to −5.8, ML = 0.4 to 0.8, DV = −7.0 to −8.5 [43]. Nine electrode tracks (separated from each other by 200 µm), with a constant stereotaxic coordinates pattern for all animals, were made in each recorded region. Extracellular recordings were performed using a single-barrel glass micropipette that was filled with 2 M NaCl (in vitro resistance 4–7 MΩ). Electrical signal of spontaneous spiking activity was measured using a high input impedance amplifier (IsoDAM-8A; WPI) and a 10–3000 Hz bandwidth filtering. The signal was digitized with a digidata 1224 (Axon Instruments) and acquired using the Pclamp software (Axon Instrument). Dopaminergic neurons were identified according to previously established electrophysiological characteristics [44]. The data are expressed as the mean active neurons per track (±S.E.M.).

### 4.9. Statistical Analysis

Microdialysis experiments and electrophysiological data which passed the Lilliefors normality test were evaluated statistically using a one-way ANOVA followed by the Newman-Keuls multiple comparisons test; otherwise, the statistical methods used non-parametric tests that are specified in figure legends. * *p* < 0.05; ** *p* < 0.01; *** *p* < 0.001.

## Data Availability

Data available on reasonable request.

## Supplementary Materials

The following are available online at https://www.mdpi.com/article/10.3390/ijms22136974/s1.

## Abbreviations

XA: xanthurenic acid; KNYA, kynurenic acid; 5-HTP, 5-hydroxytryptophan; KP, kynurenine pathway; KATs, kynurenine aminotransferase; 3-HK, 3-hydroxykynurenine; 3-HAN, 3-hydroxyanthranilic; QUIN, quinolinic acid; KMO, kynurenine monooxygenase; VTA, ventral tegmental area; SN, substantia nigra compacta; NCS-486, 4-hydroxyquinoline-2-carboxylic acid analogue; 6-OHDA, 6-hydroxydopamine; DA, dopamine.
