# Supplementary material for: A Role for Xanthurenic Acid in the Control of Brain Dopaminergic Activity
Source: Int J Mol Sci. 2021 Jun 28;22(13):6974. doi: 10.3390/ijms22136974 (PMC8268472; doi:10.3390/ijms22136974)
Supplement: Supplementary file 1 [file ijms-22-06974-s001.zip › ijms-1244323-supplementary.pdf]

Supplementary Materials file

<u>Supplementary figure S1</u>: Representative examples of chromatograms of XA and dopamine

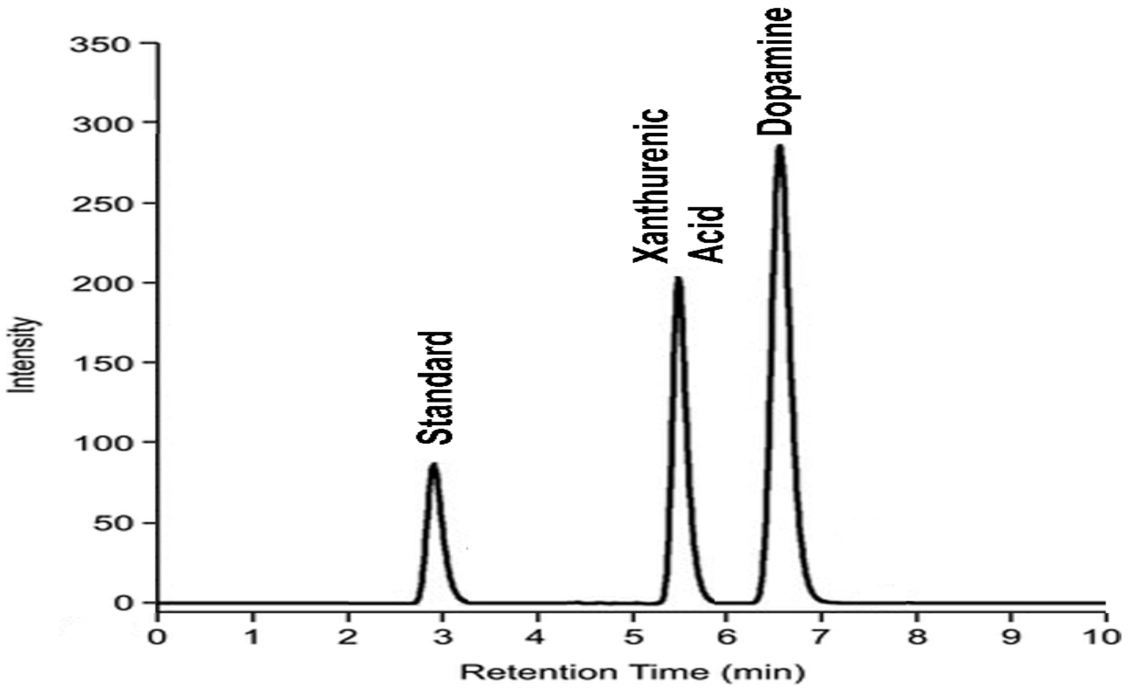

<u>Supplementary table 1</u>: Distribution of XA in some brain regions of the rat after i.p. administration

Rats (male Wistar rats, 160-180g) were injected i.p with 50 mg/kg XA (sodium salt). After 30 min, the rats were killed by microwave irradiation and their brain were dissected and homogenized in perchloric acid (10% W /v). Concentrations of XA were analyzed using HPLC. Results ± SEM (3 animals per point).

The results are in ng/mg protein. It could be noticed that the dopaminergic nuclei contain a high level of XA at physiological concentrations. Student t-test, * $p<0.05$; ** $p< 0.01$; *** $p<0.001$

Brain structure	Physiological concentrations	After i.p administration	% increase
Pre-frontal cortex	146 ± 90	1297 ± 351	9 (**)
Frontal cortex	< 50	1448 ± 165	30 (***)
Parietal cortex	416 ± 23	2565 ± 692	6 (*)
Temporal cortex	110 ± 60	995 ± 360	9 (*)
Striatum	48 ±4	595 ± 294	12 (*)
Amygdala	297 ± 13	1204 ± 180	4 (*)
Substantia nigra, VTA	397 ± 73	1307 ± 351	3 (*)

<u>Supplementary figure S2</u>: Representative examples of traces of the electrophysiological recordings for XA and Haloperidol

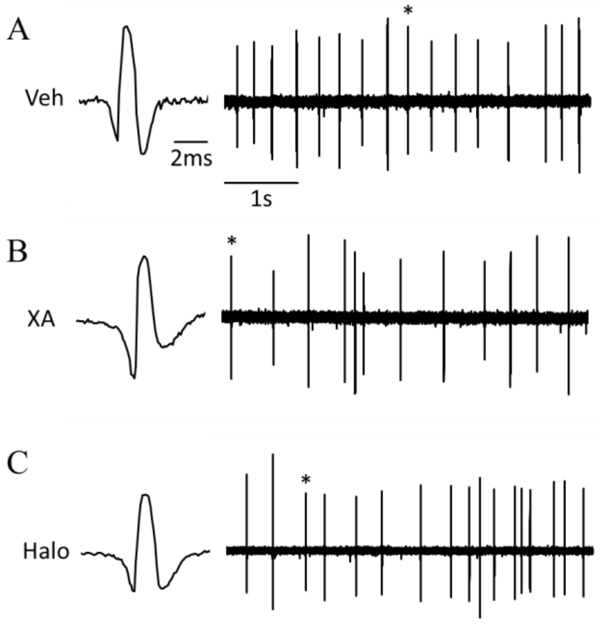

Examples of electrophysiological traces from VTA showing the spontaneously firing neurons registered from chronically vehicle- (A), XA- (B) or Halo-treated (C) rats. Individual action potentials (AP) showed (left side) are expensed traces of the APs indicated by stars in the 5s traces appearing at the right side of the image.

<u>Supplementary figure S3</u>: Recorded traces and number of events used in figure 6

Number of spontaneously firing neurons observed in SN and VTA nuclei from both left (left tracks) and right (right tracks) nuclei registered from anesthetized rats administered with either vehicle-, XA-, or Halo-rats group. The mean of left and right tracks numbers is given in the third column for SN and VTA nuclei respectively for each rat. These means are given as individual data points in figure 6. Example of traces (given on top left) corresponding to the records obtained from the left SN nucleus of the vehicle treated rat-1 for tracks 1 to 9 as indicated by the red framing and arrow. When active neurons were encountered in a track a representative trace is given for each firing neuron otherwise a baseline recorded in the silent track is given.

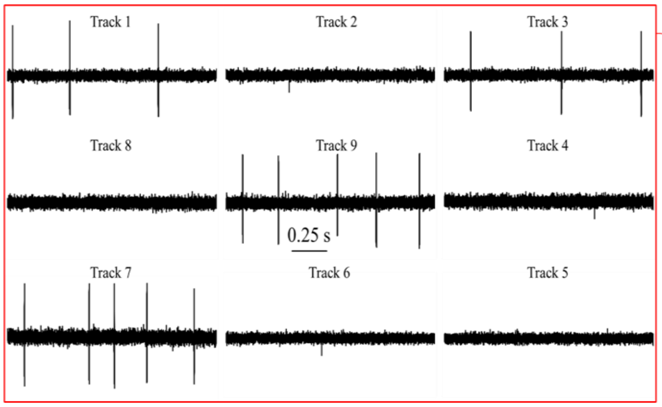

Vehicle group		Mean	Left tracks									Right tracks								
rat-1	SN	0.4	1	0	1	0	0	0	1	0	1	0	1	0	0	1	0	0	1	0
	VTA	1.4	2	1	1	1	2	1	1	2	1	1	2	1	1	1	3	1	1	2
rat-2	SN	0.5	0	2	1	0	1	0	1	0	1	1	0	0	0	1	0	0	0	1
	VTA	1.5	3	1	1	2	1	1	2	1	1	1	1	2	1	2	1	2	3	1
rat-3	SN	0.8	1	0	1	2	1	1	0	1	0	0	1	2	0	0	1	1	1	1
	VTA	1.8	3	2	1	1	2	2	1	2	2	1	2	3	1	3	2	2	2	1
rat-4	SN	0.9	1	1	1	0	2	0	1	0	1	1	2	1	1	2	0	0	1	1
	VTA	2.2	2	3	2	2	3	2	3	2	2	2	2	3	2	2	2	1	3	2
rat-5	SN	0.5	0	0	1	1	1	1	0	0	1	1	1	0	0	0	1	0	1	0
	VTA	1.7	2	1	2	2	2	1	2	2	1	2	3	1	2	1	2	2	1	2
rat-6	SN	0.5	1	0	0	0	0	1	1	1	0	0	1	0	1	1	1	1	0	0
	VTA	0.8	1	1	1	1	0	1	1	0	1	1	1	1	1	1	0	1	1	1

XA group		Mean	Left tracks									Right tracks								
rat-1	SN	1.2	2	1	1	1	0	1	2	1	1	1	1	2	1	1	1	1	2	1
	VTA	1.2	1	1	2	1	2	1	1	1	1	2	1	1	2	1	1	1	1	1
rat-2	SN	0.7	0	1	1	1	1	1	1	1	0	1	2	0	0	1	0	1	1	0
	VTA	1.0	1	1	1	1	1	1	1	1	1	1	1	1	1	1	1	1	1	1
rat-3	SN	0.6	1	0	1	1	0	1	0	1	0	0	1	1	0	0	1	1	0	1
	VTA	0.7	1	1	0	1	1	1	0	1	1	1	1	0	1	1	1	0	0	1
rat-4	SN	1.0	1	2	1	2	1	0	1	0	1	1	0	1	1	1	1	2	1	1
	VTA	0.9	1	0	1	1	1	0	1	2	1	1	0	1	1	1	0	1	1	2
rat-5	SN	0.6	0	1	0	0	0	2	1	0	2	0	1	1	1	1	0	0	0	1
	VTA	0.8	0	1	1	1	1	1	1	0	1	1	1	1	1	0	1	2	1	0
rat-6	SN	0.6	1	0	0	0	0	1	1	1	0	0	1	0	1	1	1	1	0	1
	VTA	0.8	1	0	1	1	1	0	1	1	1	1	1	1	1	0	1	1	0	1

Halo group		Mean	Left tracks									Right tracks								
rat-1	SN	0.1	1	0	0	0	0	0	0	0	0	0	1	0	0	0	0	0	0	0
	VTA	0.4	0	0	0	1	0	1	1	1	0	1	0	1	0	0	0	1	1	0
rat-2	SN	0.2	0	0	1	0	0	1	0	0	0	1	0	0	0	1	0	0	0	0
	VTA	0.5	0	0	1	0	1	1	0	0	1	0	1	0	1	1	1	0	0	1
rat-3	SN	0.4	0	0	1	0	0	1	0	1	0	0	1	0	0	0	1	1	0	1
	VTA	1.2	2	1	1	1	1	1	2	1	1	1	1	3	1	1	1	2	0	1
rat-4	SN	0.3	0	1	0	0	1	0	0	0	1	0	0	0	1	0	0	0	1	1
	VTA	0.8	1	1	1	0	1	1	1	1	1	1	1	1	0	1	1	0	1	1
rat-5	SN	0.2	0	0	0	0	0	0	1	0	0	0	0	1	0	1	0	0	0	1
	VTA	0.6	0	1	0	1	1	0	1	0	1	1	0	1	1	1	0	1	1	0
rat-6	SN	0.1	0	0	0	0	0	1	0	0	0	0	0	0	0	0	0	0	1	0
	VTA	0.6	1	0	1	0	1	0	1	1	0	1	1	1	1	0	0	1	0	1

<u>Supplementary figure S4:</u> Peripheral XA administration to rats modifies some behavioral parameters.

Several behavioral parameters were explored in rats after peripheral XA administration. The most significative results are given below.

S4A Modification of locomotor activity as a function of XA doses

Increased doses of XA (sodium salt) were injected was I.P route to Wistar rats (from 12.5 to 200 mg/kg). Spontaneous locomotor activities were recorded by infra-red light. The figure below represents the decrease of motor activities for XA doses above 50mg/kg. This decrease augments with the dose of XA (3 rats were used for each experimental point).

Anova test : ** $p<0.01$; *** $p< 0.001$.

S4B Behavioral despair tests in rats

This test was carried out on male Wistar rats weighing 160-180g receiving a sub-chronic dose of XA (i.p administrations of 50, 100, 150 et 200 mg/kg. The reference antidepressant drug was imipramine 30mg/kg). This test measures the immobility of the animals in a forced swimming situation. Immobility was reduced by various clinically effective antidepressant drugs, like imipramine. This test is suggested to reveal antidepressant effect of drugs (Porsolt, R.D., Eur. J. Pharmacol. 47 (1978) 379-391).

XA and imipramine were administered as a series of 3 i.p injections 24, 5 and 1h before the 5 min immobility test in the swimming plexiglass cylinder, as described by Porsolt et al. XA doses of 100, 150 and 200 mg/kg have shown an activity close to those of imipramine.

Anova test ** $p<0.05$; *** $p< 0.01$.

S4A

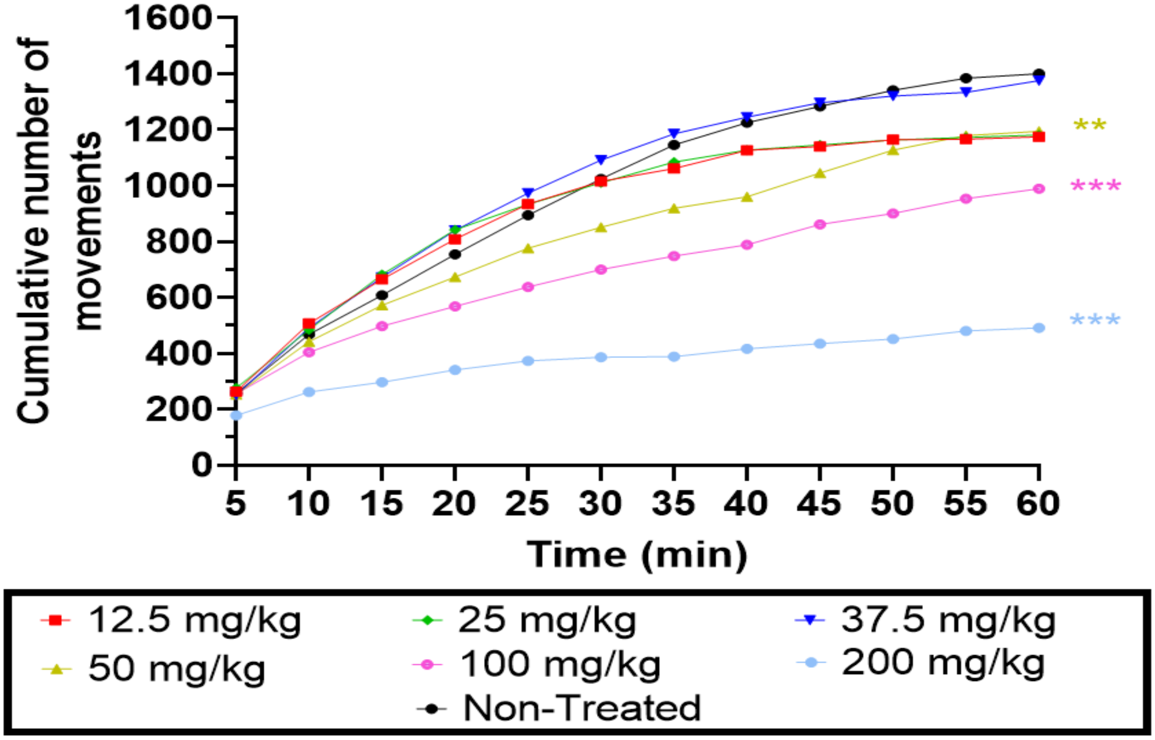

Cumulative number of movements
1600
1400
1200
1000
800
600
400
200
0
5
10
15
20
25
30
35
40
45
50
55
60
Time (min)
**
***
***
12.5 mg/kg
25 mg/kg
37.5 mg/kg
50 mg/kg
100 mg/kg
200 mg/kg
Non-Treated

S4B

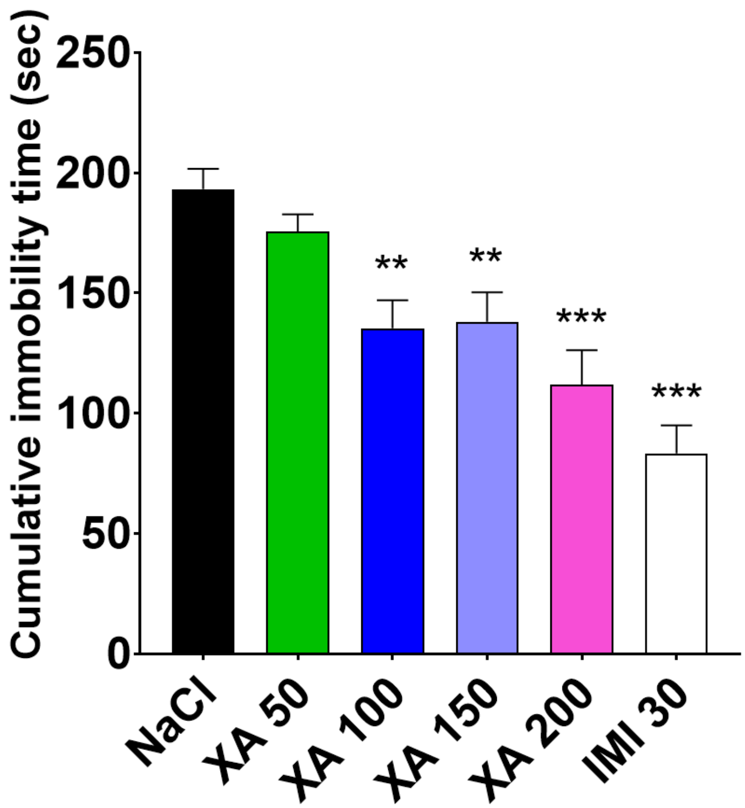
Cumulative immobility time (sec)
250
200
150
100
50
0
**
**
***
***
NaCl
XA 50
XA 100
XA 150
XA 200
IMI 30
